# A hybrid Neural Network-SEIR model for forecasting intensive care occupancy in Switzerland during COVID-19 epidemics

**DOI:** 10.1371/journal.pone.0263789

**Published:** 2022-03-03

**Authors:** Riccardo Delli Compagni, Zhao Cheng, Stefania Russo, Thomas P. Van Boeckel

**Affiliations:** 1 Health Geography and Policy Group, ETH Zürich, Zürich, Switzerland; 2 Ecovision Lab, Photogrammetry and Remote Sensing, ETH Zürich, Zürich, Switzerland; 3 Center for Diseases Dynamics Economics and Policy, Washington, DC, United States of America; Indian Institute of Technology Patna, INDIA

## Abstract

Anticipating intensive care unit (ICU) occupancy is critical in supporting decision makers to impose (or relax) measures that mitigate COVID-19 transmission. Mechanistic approaches such as Susceptible-Infected-Recovered (SIR) models have traditionally been used to achieve this objective. However, formulating such models is challenged by the necessity to formulate equations for plausible causal mechanisms between the intensity of COVID-19 transmission and external epidemic drivers such as temperature, and the stringency of non-pharmaceutical interventions. Here, we combined a neural network model (NN) with a Susceptible-Exposed-Infected-Recovered model (SEIR) in a hybrid model and attempted to increase the prediction accuracy of existing models used to forecast ICU occupancy. Between 1^st^ of October, 2020 - 1^st^ of July, 2021, the hybrid model improved performances of the SEIR model at different geographical levels. At a national level, the hybrid model improved, prediction accuracy (i.e., mean absolute error) by 74%. At the cantonal and hospital levels, the reduction on the forecast’s mean absolute error were 46% and 50%, respectively. Our findings illustrate those predictions from hybrid model can be used to anticipate occupancy in ICU, and support the decision-making for lifesaving actions such as the transfer of patients and dispatching of medical personnel and ventilators.

## 1. Introduction

On March 11^th^, 2020, the World Health Organization (WHO) declared the COVID-19 pandemic an international health emergency [1]. Since then, COVID-19 has caused infections in millions of people [2], with a substantial proportion of infections (e.g. 9–11% [3]) requiring hospitalization in intensive care units (ICU). In multiple countries, demand of ICU beds exceeded bed availability [4–6], leading to excess mortality of COVID-19 patients as well as backlogs of patients for other pathologies that require hospitalization in ICU [7–9]. Monitoring and anticipating ICU occupancy has become critical to support decision-makers to impose (or relax) non-pharmaceutical interventions that can help mitigate the transmission of COVID-19, and thereby reduce its impact on healthcare systems.

Mathematical models have been used extensively to anticipate the evolution of epidemic indicators, including the occupancy of ICU [10–13]. In particular, two families of mathematical models have been predominantly used: 1) mechanistic models (MMs), including Susceptible-Infected-Recovered (SIR) models [14] and their extensions into agent-based models [15], as well as, 2) statistical approaches [16], including machine learning (ML) models [17]. Each family of model present advantages and disadvantages: MMs typically consist of differential equation systems that reflect biological mechanisms that govern the dynamic of infections. The parameters of these equations usually have a biological meaning (i.e., an infectious period) and therefore can be used for predictions outside of their calibration space (i.e., scenario analysis). However, for MM, accounting for the causal mechanisms between ICU occupancy and environmental covariates (e.g. changes in environmental conditions [18,19]) comes at the cost of additional parameters to be estimated in a differential equations system. In contrast, ML models seek to establish statistical associations between response variables and potential covariates without making assumptions about potential biological mechanisms [20]; however, because ML models are based on statistical associations and not causation, their validity is bound to their calibration space, and every prediction outside such a space can lead to inconsistent results [21,22]. Therefore, the combination of MMs and ML models in “hybrid models” has been explored in a variety of fields [21–23] (e.g., earth systems, climate science, biology, hydrology, etc.), and have showed promising results for improving prediction accuracy [20] from MM models. One of the most common configurations for a hybrid model is known as “residuals modelling”, and is of particular interest when the MM formulation may be too limited to capture complex associations between a response variable and its covariates [23]. Concretely, this configuration consists of using a MM to capture the overall temporal trend of a temporally autocorrelated process while letting the ML model compensate for any residual error that is potentially associated with external drivers of the process of interest. Neural Networks (NN) are one of the most commonly used ML models in this framework due to their ability to implicitly capture nonlinearities and interactions [24]. MMs have been coupled with NNs in different fields, thereby improving performances of the corresponding MMs: for example, Chu et al. [25] improved prediction accuracy of a MM to simulates performances of a centrifugal compressor; Lee et al. [26] also improved prediction accuracy of a MM to simulate the operations of a waste-water treatment plant; Thompson and Kramer [22] used a NN to model a fed-batch penicillin fermentation reaction. Thus far, multiple works have shown how hybrid models can be used to predict the evolution of the COVID-19 epidemic [27–29]; however, to the best of our knowledge, these works did not implement the configuration of residual modelling using a NNs as a ML model.

In this study, we developed a hybrid model based on the residual modelling configuration aimed at increasing the prediction accuracy of an SEIR model (Susceptible-Exposed-Infected-Recovered) across spatial scales for producing short-term (3- and 7-days ahead) predictions of ICU occupancy. The accuracy of the modelling framework was tested in Switzerland, where data on ICU occupancy were available at different geographical levels (i.e., national, cantonal, hospital). Finally, we also downscale predictions of the hybrid model at the hospital-level to support hospital management actions.

## 2. Materials and methods

### 2.1. Mechanistic model (MM)

We used the SEIR model described in Zhao et al. [30]. to simulate the dynamics of occupancy of ICU from the 6^th^ of November 2020 until 1^st^ of July 2021. This model was expanded to include the impact of vaccination campaigns [31]. This period included three epidemic phases: phase 1, from the lockdown (19^th^ of October, 2020) until the start of second-dose vaccinations (15^th^ of January, 2021); phase 2, from the start of second-dose vaccinations until the relaxation (14^th^ of April, 2021); and phase 3, from the relaxation until 1^st^ of July, 2021. The phases are reported in S1 Fig in the Supplementary Information (SI).


$$\beta =\frac{R_{0}}{N}\times k\times \gamma ,$$

$$\frac{dS(t)}{dt}=-S\beta I-c,$$

$$\frac{dE(t)}{dt}=+S\beta I-\sigma E,$$

$$\frac{dI(t)}{dt}=+\sigma E-\gamma I,$$

$$\frac{dP(t)}{dt}={+\varepsilon }_{1}\gamma I-\omega_{1}P$$

$$\frac{dH_{1}(t)}{dt}=+\omega_{1}P-\omega_{2}H_{1},$$

$$\frac{dH_{2}(t)}{dt}=+{(1-\varepsilon }_{2})\omega_{2}H_{1}-\omega_{3}ICU,$$

$$\frac{dICU(t)}{dt}=\varepsilon_{2}\omega_{2}H_{1}-{(1-\varepsilon }_{4})\omega_{4}ICU-\varepsilon_{4}\omega_{5}ICU,$$

$$\frac{dR(t)}{dt}=+{(1-\varepsilon }_{1})\gamma I+{(1-\varepsilon }_{3})\omega_{3}H_{2}+{(1-\varepsilon }_{4})\omega_{4}ICU,$$

$$\frac{dD(t)}{dt}={+\varepsilon }_{3}\omega_{3}H_{2}+\varepsilon_{4}\omega_{5}ICU,$$

$$\frac{dC(t)}{dt}=+\gamma I.$$

Where *S* (Susceptible), *E* (Exposed), *I* (Infected), *P* (infected but not yet hospitalized), *H* (= *H*_1_ + *H*_2_, Hospitalized), *ICU*, *D* (Death), *R* (Recovered), and *C* (Cumulative Infected) are the model variables; *R*_*0*_ the basic reproduction number, *c* the vaccination rate, and *k* the reduction/increase in transmission rate after a non-pharmaceutical intervention is introduced/relaxed. Parameter values and their meanings are reported in Table 1. Daily vaccination rates were obtained from the public dashboard of the Swiss Federal Office of Public Health [32].

**Table 1:** Model parameters of the SEIR model (adapted from Zhao et al. [30]).

Parameter	Description	Value
*R*_0_	Basic reproduction number	Estimated
*κ*	Percentage of *R*_0_	Estimated
c	Vaccination rate	Estimated
*σ*, *γ*	Serial interval	1/2.6 days [33]
*ω*_1_	Duration from onset of symptoms to hospitalization	1/5 days [34]
*ω*_2_	Initial hospitalization	1/6 days [34]
*ω*_3_	Additional days of hospitalization until recovery/death	1/10 days [34]
*ω*_4_	Additional days in ICU until recovery	1/13.1 days *
*ω*_5_	Additional days in ICU until death	1/12.7 days *
*ε*_1_	Rate of *H* admission of infected	0.0161 [35]
*ε*_2_	Hospitalized cases requiring critical care in ICU	30% [34]
*ε*_3_	Death outside of ICU	35% [36]
*ε*_4_	Death rate from ICU	22%*

* Obtained for patients (n = 382) included in the RISC-19-ICU registry supported by Swiss Society of Intensive Care Medicine (https://www.sgi-ssmi.ch).

### 2.2. Machine learning model

#### 2.2.1. Model structure

**Fig 1:**
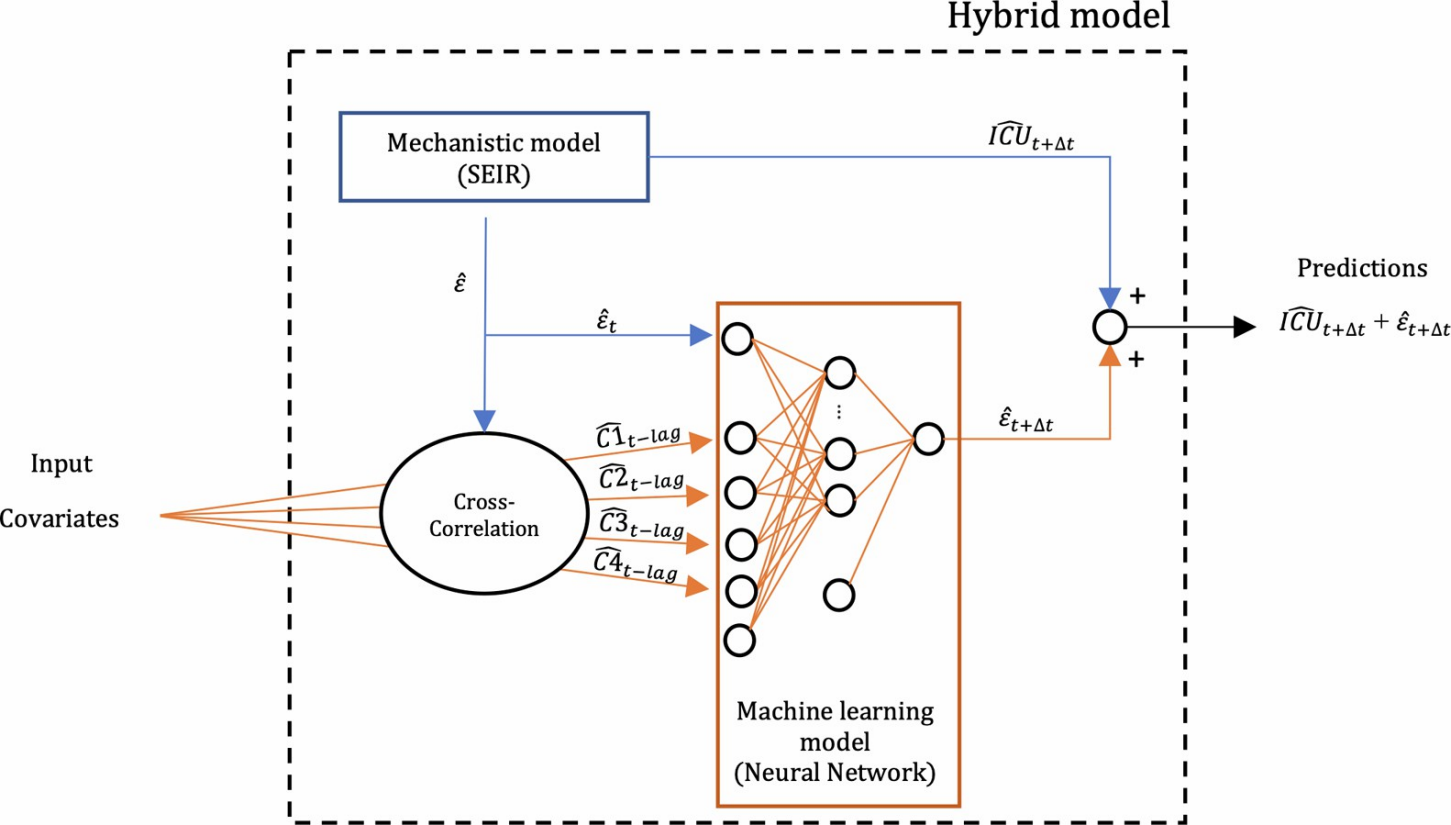
Configuration of the hybrid model. The hybrid model combines a mechanistic model (SEIR) with a machine learning model (Neural Network).

We used a feed-forward NN with a single hidden layer [37,38] to predict the residuals (${\hat{\varepsilon }}_{t+\Delta t)}$) of the SEIR model (Fig 1). This choice was based on two properties that make this type of NN suitable for our purpose: first the ability to account for nonlinearities and interactions between response variables and covariates [24,39]. Second, ability of NN models to be trained on relatively small datasets that is comparatively higher than for other ML models such as deep neural networks [40].

Covariates (section 2.2.2.) were introduced in the NN with a lag corresponding to the maximum correlation with the response variable via cross-correlation. In particular, the lag of *t—*Δ*t*,…, *t– 42* was explored among the possibilities, with 42 days as the minimum value. An additional covariate, ε_*t*_, was also added to account for the autoregressive nature of process.

The NN formulation was:

$${\hat{\varepsilon }}_{t+\Delta t}=T\left(\omega_{0}+\sum_{j=1}^{J}\omega_{j}∙T\left(\omega_{0j}+w_{j}^{T}x\right)\right)$$ (1)

Where Δ*t* was set to 3 or 7 days; *ω*_*0*_ is the intercept of the output layer, and *ω*_*0j*_ the intercept of *j*^th^ hidden node; *ω*_*j*_ is the weight (also known as parameter) associated with the connection from the *j*^th^ hidden node to the output layer, and w_j_^*T*^ is the vector of weights associated with the connection to the j^th^ hidden node; Γ is the Rectified Linear Unit (ReLU) activation function; *x* is the vector of covariates. The size of the of the hidden layer, determined by the number of hidden nodes, was optimized together with other hyperparameters. Specifically, hyperparameters are different from parameters: parameters are learned during model training, while hyperparameters need to be optimized externally to model training (see section 2.3.).

#### 2.2.2. Covariates

These covariates were used to predict ICU occupancy (Table 2): i) the number of COVID-19 cases; ii) the number of COVID-19 cases associated with the Alpha variant (better known as UK variant); iii) the level of non-pharmaceutical interventions (e.g., school closures, workplace closures, and travel bans) as identified by the Containment and Health Index (i.e., a subindex of the Stringency Index [41]); and iv) the mean daily air temperature.

**Table 2:** List of covariates.

Name	Source ^Reference^
COVID-19 cases	Open Swiss Government data set [42]
Proportion of COVID-19 cases associated to the Alpha variant	Github repository [43]
Index of Containment and Health	Github repository [44]
Mean environmental temperature	opendata.swiss [45]

### 2.3. Model training and performance evaluation

**Fig 2:**
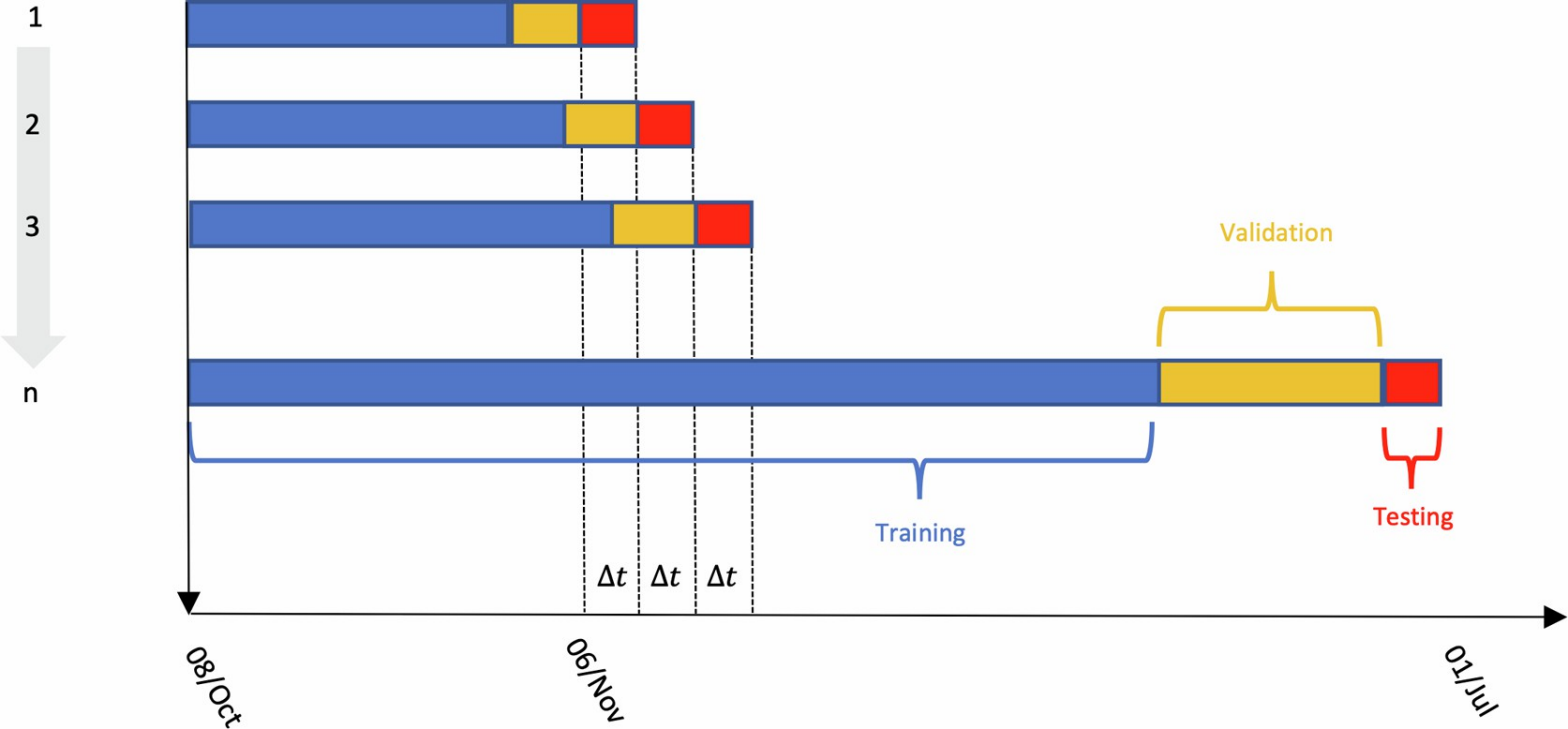
Temporal cross-validation scheme. Δt was set to 3 and 7 days for the predictions at 3 and 7 days ahead, respectively.

We adopted a temporal cross-validation scheme [46] similar to the one used by Vollmer et al [29]. This scheme (Fig 2) allowed us to train and evaluate the performance of the hybrid model multiple times (n = 85 for the prediction at 3-days and n = 36 for the prediction at 7-days) over the simulated period.

The scheme works as follows:

*First*, the time series is divided in three successive time windows: the training set, validation set, and test set (Fig 2). The training and the validation sets are used for the optimization of the parameters of the SEIR model (one *κ*, the reduction applied to R_0_, estimated separately for of the 3 phases), as well as the optimization of hyperparameters of the NN (i.e., the number of nodes in the hidden layer, learning rate, and dropout rate). A training-validation split of 90%/10% is adopted. The initial training and validation set included data from the 8^th^ of October, 2020 until the 6^th^ of November, 2020, in order to meet a minimum amount of data for model training.

*Second*, the performance of the trained model is evaluated on the test set using the mean absolute error. The test set consisted of n = Δ*t* values, with Δ*t* equals to 3 and 7 for the predictions at 3- and 7-days ahead, respectively.

*Third*, the training-validation set is expanded to include the test set of the previous iteration.

At the end of the iterative validation scheme, the overall performance of each model is estimated using the average MAE across iterations on the successive test sets (average MAE on red block for iteration 1 to n Fig 2).

In step 2, the optimization of the SEIR model was performed using maximum likelihood (Nelder and Mead algorithm [47]) on the complete training-validation set; residuals of the SEIR model are then calculated. The optimization of the hyperparameters of the NN was done as follows: a sampling space of 100 combinations of hyperparameters was generated using a Latin Hypercube [48]. A back-propagation algorithm (based on gradient descent) was used as a learning algorithm to modify the values of the weights and obtain the best matches possible between the true and estimated values of the residuals of the SEIR model in the training set. The mean absolute error (MAE) was used as fitting criteria on the validation set, and an early stopping mechanism was applied to stop the learning algorithm if the MAE did not achieve a decrease of 5 units within 100 epochs (i.e., number of iterations that the learning algorithm worked through the training set). The largest number of possible epochs was set to 4,000. We accounted for the stochastic nature of the optimization by repeating the simulation 10 times for each combination of hyperparameters. For each set of 10 simulations, we calculated the mean and standard deviation of the MAE in the validation set. The combination of hyperparameters that generated the minimum mean MAE in the validation set was selected as optimal for the NN.

Performance evaluation was evaluated on the test set as follows: i) predictions of the SEIR model (${\hat{ICU}}_{t+\Delta t)}$) were based on the parameters inferred in the training-validation set (extrapolation); ii) predictions of the NN (${\hat{\varepsilon }}_{t+\Delta t)}$) were obtained after training a NN with the optimal combination of hyperparameters on both the training and validation set; iii) the sum of the two contributions (${\hat{ICU}}_{t+\Delta t)}+{\hat{\varepsilon }}_{t+\Delta t)}$) was compared with the observed ICU occupancy. Confidence intervals for the NN were generated using the standard deviation calculated on the validation set, while they were obtained as described in Zhao et al. for the SEIR model [30], with the 2.5% and 97.5% quantiles of the 10,000 predictions.

Furthermore, the predictions (and accuracy evaluated via the MAE) of the hybrid model were compared to that of the SEIR and NN model independently.

### 2.4. Downscaling at hospital level

Model predictions were obtained at the national- and cantonal-level, and from the cantonal-level downscaled to the hospital-level. Particularly, cantonal-level predictions were downscaled based on the percentage of occupancy of ICU beds in each hospital, calculated as moving average of the past Δ*t* days. The ICU occupancy data of Swiss hospitals were provided daily by the Coordinated Sanitary Service of the Swiss Armed Forces, and refer to the number of ICU beds occupied by COVID-19 patients. The canton of Zurich was selected for model testing, since it is the most populated Canton in Switzerland with 15 hospitals with ICU.

### 2.5. Importance of covariates

We determined the relative importance of each covariate in making predictions for the hybrid model. Concretely, we computed a Deviation metric (2) between the MAE of the full model including n = 5 covariates with that of a reduced model with n = 1 covariates [49]. The Deviation was calculated on the test set at the end of each epidemic phase. The procedure was repeated n = 5 times excluding one covariate at a time. The Deviation (%) was calculated as follows:

$$Deviation=\frac{{MAE}_{Reduced\;Model}-{MAE}_{Full\;Model}}{{MAE}_{Full\;Model}}x\;100$$ (2)

A positive Deviation signifies that the excluded covariate was important for the model. Specifically, a positive Deviation corresponds to a decreased accuracy of the reduced model compared to the accuracy of the full model that included all covariates.

## 3. Results

### 3.1. Model comparison

**Fig 3:**
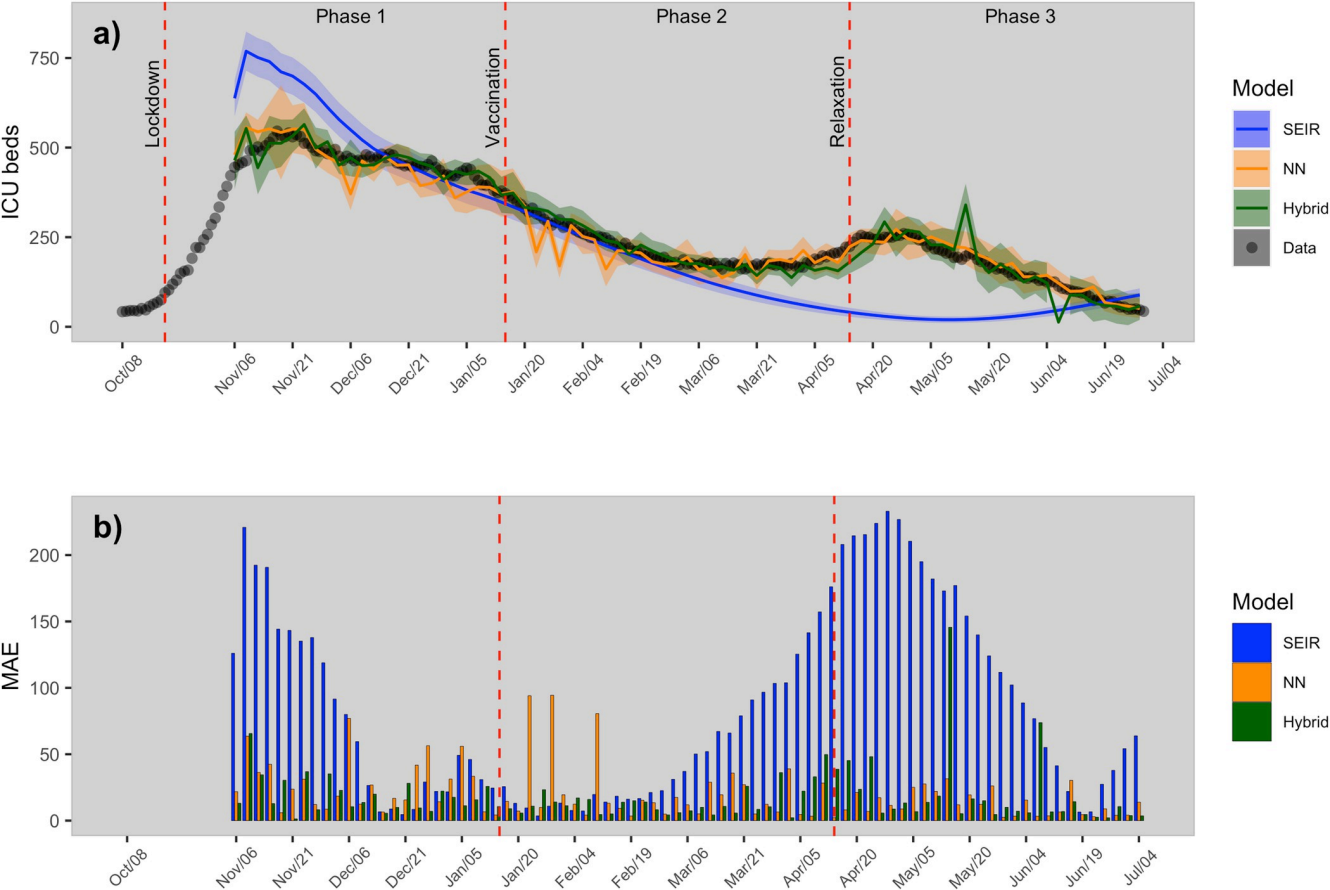
Model predictions of intensive care bed occupancy at the national-level. a) Predictions 3-days ahead of intensive occupancy at the national-level for the three models (shaded areas represent 95% confident intervals); b) corresponding Mean Absolute Error (MAE) calculated on test data.

We compared 3 types of epidemic models (i.e., SEIR, NN and hybrid,) to predict short-term (3 and 7 days ahead) ICU occupancy at the national- and cantonal-level. Fig 3 shows the 3-day predictions at the national-level from the three models (a), and with its associated MAE calculated on the test set (b).

During phase 1 (19^th^ of October, 2020 - 15^th^ of January, 2021) the hybrid model (average MAE = 19 beds) outperformed both the NN (average MAE = 27 beds) and the SEIR model (average MAE = 78 beds). During phase 2 (15^th^ of January, 2021 - 14^th^ of April, 2021), the hybrid model remained the most accurate model (average MAE = 16 beds), although the performance of the NN (average MAE = 21 beds) and the SEIR model (average MAE = 59 beds) improved in comparison with phase 1.

During phase 3 (14^th^ of April, 2021 - 1^st^ of July, 2021), the hybrid model (average MAE = 19 beds) was slightly outcompeted by the NN (average MAE = 13 beds); while the SEIR model was associated with the worse performances (average MAE = 125 beds). S1 Fig in Supporting Information (SI) showed predictions at 7-days ahead and its corresponding MAE.

**Fig 4:**
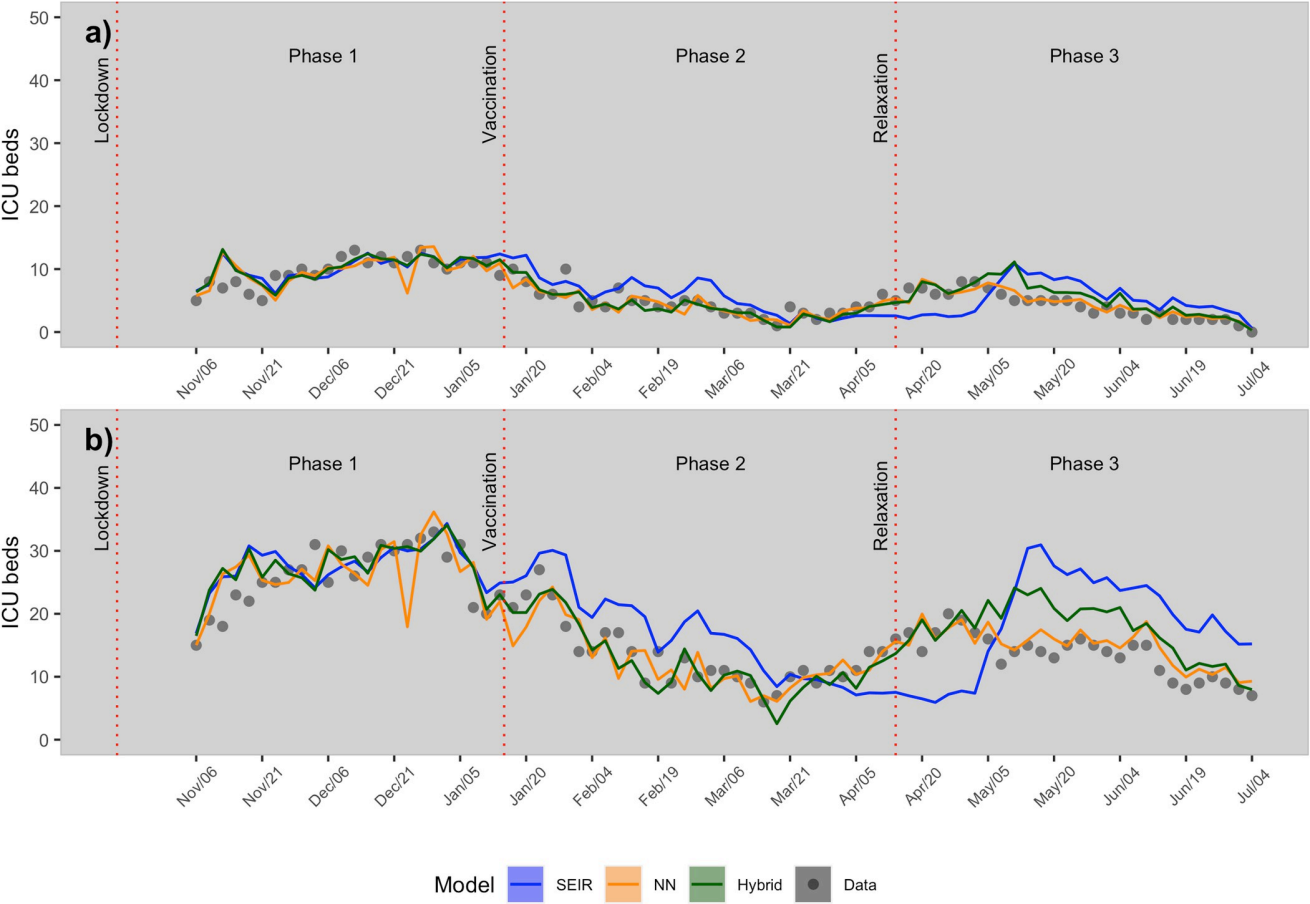
Model predictions of intensive care bed occupancy at the hospital-level. Prediction at the hospital-level for a medium-sized hospital (a) and the largest hospital (b) in the canton of Zurich.

Predictions 3-days ahead (S2 Fig) were then downscaled from the cantonal-level to the hospital-level. Results for a medium-sized hospital, as well as the biggest hospital in the canton of Zurich are shown in Fig 4. At the hospital-level, the hybrid model outperformed the SEIR model for both the medium-sized hospital (average MAE_hybrid_ = 1.2 beds, average MAE_SEIR_ = 2.2 beds) and largest hospital (average MAE_hybrid_ = 3.1 beds, average MAE_SEIR_ = 6.2 beds) in the canton of Zurich. In comparison, the NN model performed on average as good as the hybrid model for both hospitals. Similar to the national-level scenario, the highest average MAE for the SEIR model was observed during phase 3, during which the SEIR model was not capable of capturing the occupancy increase of ICU that occurred two months after the start of vaccination (15^th^ of January, 2021).

### 3.2. Relative importance of covariates

**Fig 5:**
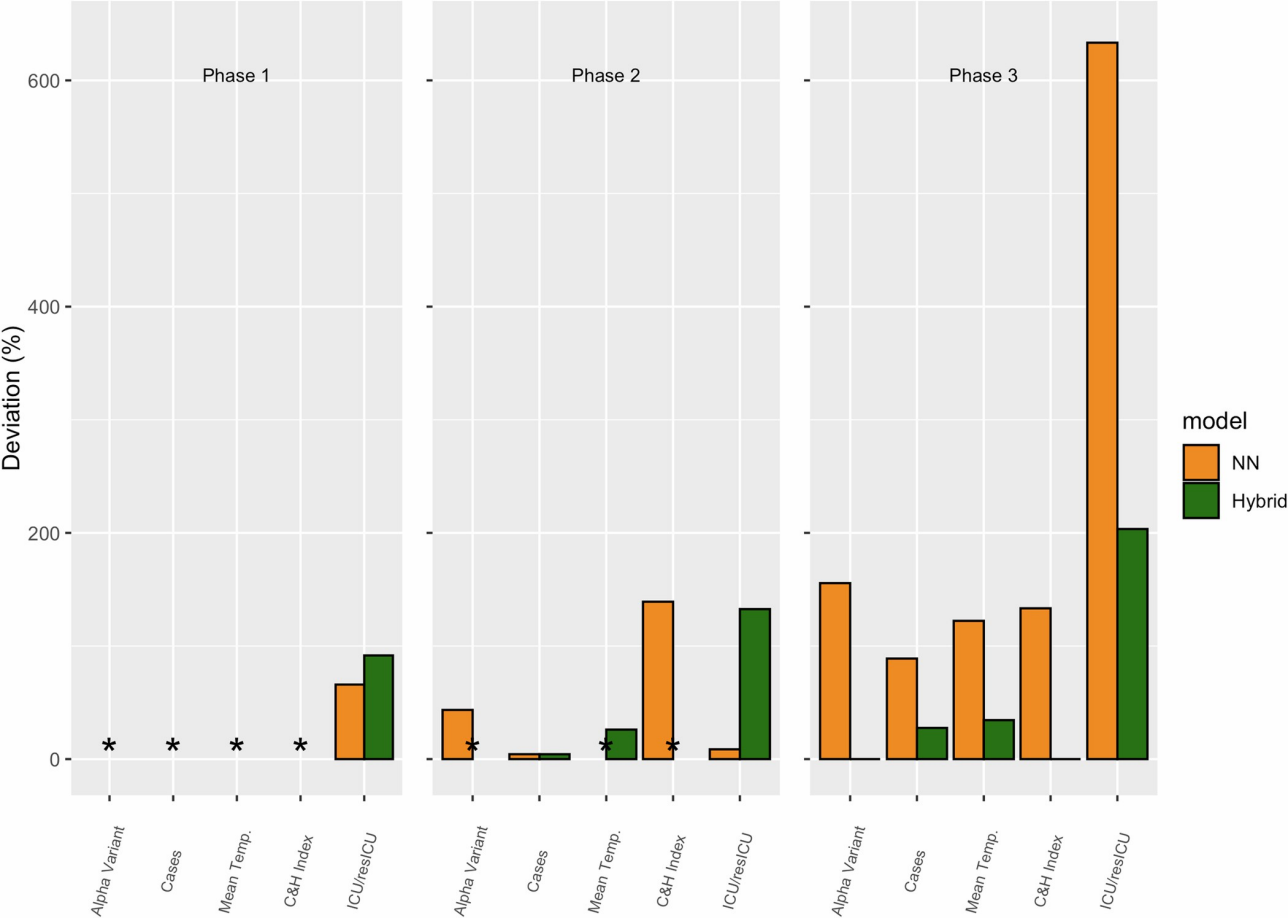
Covariance importance for each phase. An asterisk represents a negative deviation.

The relative importance of covariates during each of the three phases is reported in Fig 5. In phase 1, a negative Deviation (marked with an asterisk in the Figure) was observed for a majority of the covariates (i.e., COVID-19 cases, proportion of COVID-19 cases associated to the alpha variant, Index of Containment and Health, and mean environmental temperature), meaning that their exclusion from the full model improved prediction accuracy. Conversely, the autoregressive covariate was important for making predictions, with Deviations equal to 92% and 66% for the hybrid and NN model, respectively. In phase 3, all of the covariates were informative; in this last phase, the NN predictions were more affected by the exclusion of covariates in comparison to the hybrid model. The average Deviation was 230% and 53%, for the NN and hybrid model, respectively.

## 4. Discussion

### 4.1. Prediction accuracy

In this study, we showed increased prediction accuracy of ICU occupancy using a hybrid model combining a SEIR and a NN model. The model developed here could help guide interventions against future COVID-19 epidemics. At a national-level, during phase 1 (19^th^ of October, 2020 - 15^th^ of January, 2021) the overestimation of ICU occupancy by the SEIR model could be associated with its intrinsic nature to predict exponential growth at the beginning of a new wave. In contrast, the SEIR model underestimated the ICU occupancy during phase 3 (14^th^ of April, 2021 - 1^st^ of July, 2021). This could be explained by the fact that the model lacks important covariates such as temperature, which may have been responsible of an increase of cases during the winter and thus for an increased ICU occupancy. For the NN model, its worst performance was observed during phase 1, where abrupt oscillations occurred. These oscillations could be attributed to the short time series available for model training at that stage, thereby compromising model training and limiting predictive performance. This interpretation is supported by the fact that the prediction accuracy of the NN model improved during phase 2 (15^th^ of January, 2021 - 14^th^ of April, 2021) and 3 (14^th^ of April, 2021 - 1^st^ of July, 2021), when longer time series became available for model training.

At the national- and cantonal-levels, the SEIR model was unable to capture the increase in ICU occupancy that occurred two months after the beginning of the second-dose vaccination campaign. The causal mechanisms behind this trend remains unclear, but may be associated with other drivers such new variants (e.g., Delta variant) that are not incorporated in the SEIR model. In contrast, the hybrid and NN model could capture this trend, suggesting that both models succeeded in learning potential non-linear relationships between covariates and occupancy of ICU.

### 4.2. Relative importance of covariates

The fact that the relative importance of each covariate for our models changed between phases has multiple possible interpretations. The first is that a covariate is important for making predictions during one phase, while it is not important for another phase. For example, the proportion of the Alpha variant was not informative during phase 1 when its prevalence was < 10% of the total confirmed COVID-19 cases, while it was informative during phase 3, when its prevalence was > 50% of the total confirmed Covid-19 cases. The second reason could be associated to the length of the time series. For example, the model had limited data for training during phase 1, while the amount of data tripled for phase 3. This could have caused the full model (i.e., with 5 covariates) to perform worse than the reduced model (i.e., with 1–5 covariates) [50,51], leading to negative Deviation.

During phase 1, the autoregressive term was the only informative covariate, meaning that the models behaved similarly to an Automatic Regressive Integrated Moving Average (ARIMA) model. Furthermore, on average, the Deviation associated with the hybrid model was always lower than the one associated to the NN. This means that the hybrid model was more robust in the exclusion of a specific covariate compared to the NN.

### 4.3. Possible applications and limitations

In Switzerland, a number of studies have focussed on providing long-term (>2 weeks) [52–54] and short-term (<2 weeks) [36] predictions using MMs. Predictions have predominantly on the national scale; while many of the lifesaving actions (e.g., transfer of patients) need to be planned at the cantonal- (provincial-level) or hospital-level. In this study, we attempted to increase the prediction accuracy of a SEIR model by coupling it with a NN, generating a so-called hybrid model. Among all the possible ways to combine a MM with a ML model, we opted for a configuration called residual modelling. In particular, we used a SEIR model for predicting occupancy of ICU beds under future scenarios at different geographical levels (national, cantonal, and hospital) in Switzerland; we trained a NN to supplement these predictions using the information embedded in covariates (temperature, etc…). This modelling framework could be applied in other geographic regions for which a MM (e.g., of the SIR family), and spatially explicit covariates are available. Specifically, different extension of the SIR model [14] can be used, from simple examples (like the SEIR used in this study), to increasingly complex frameworks such as SIDARTHE [12]. As for the ML model, we used a feed-forward NN with a single hidden (see section 2.2.1.). However, alternative formulations could have been chosen. For example, Maher Ala’raj et al. [27] coupled an ARIMA model, a very popular ML model for time series forecasting with a SEIRD model; Watson et al. [17] embedded a Bayesian time series model and a random forest algorithm within a SIRD model; Rahmadani and Lee [28] combined a deep-learning algorithm with a SEIR model.

As with any modelling study, our analysis also comes with limitations. For example, training of the NN is often computationally intensive and the selection of optimal hyperparameters is based on empirical rules such as try-and-error approaches [46]. In this study, the optimization of hyperparameters required a significant effort in terms of computational cost. Specifically, all simulations were run in parallel on ETH High Performance Computing facilities (Euler cluster) [55], requiring, on average, 1 minute per simulation on one CPU, and thus 15 CPU minutes for each iteration running simultaneously on 15 CPU cores. We optimized three hyperparameters, namely the number of nodes in the hidden layer, learning rate, and dropout rate; however, other hyperparameters such as the type of activation function, the number of batches, the number of epochs, etc., could also have been subjected to optimization. Furthermore, other type of search algorithms such as the sequential model-based optimization (SMBO, also known as Bayesian optimization) [56] could have been explored. Another drawback of the residual modelling configuration is the inability to enforce real-world constraints (e.g., ICU beds ≥ 0), since the residuals are modelled instead of based on the actual ICU occupancy. One possible alternative could be to combine the SEIR model and the NN in series. In this case, the NN estimates intermediate variables to be used in the SEIR model, although it would impose structural changes on the SEIR model based on the variables selected, which may be challenging to implement.

As for the downscaling at the hospital-level, we used a simple method based on the moving average to downscale predictions at the cantonal-level (see section 2.4.), demonstrating a satisfactory degree of accuracy in hospitals in the Canton of Zurich. However, this method requires the availability of ICU beds at the hospital-level, which is not always the case. Consequently, more complex methods could be tested. In particular, Zhao et al. [30] presented a method to distribute ICU patients based on travel time from the location of the patient to the hospital. In the future, our modelling framework can be updated as growing knowledge is gained on the covariates associated with the spread of COVID-19. For example, new covariates such as other virus variants and mobility patterns in different regions (e.g., people coming in and out of Switzerland) could be included to improve predictions. Lastly, the framework could be applied to improve predictions of other infectious diseases, for which a MM already exists.

## Supporting information

S1 Fig Predictions 7-days ahead of intensive occupancy at the national-level. a) Predictions 7-days ahead of intensive occupancy at the national-level for the three models (shaded areas represent 95% confident intervals); b) corresponding Mean Absolute Error (MAE) calculated on test data.
(PDF)

S2 Fig Predictions 3-days ahead of intensive occupancy at cantonal level. a) Predictions 3-days ahead of intensive occupancy at cantonal level (canton of Zurich) for the three models (shaded areas represent 95% confident intervals); b) corresponding Mean Absolute Error (MAE) calculated on test data.
(PDF)
