# Supplementary figures and images for: A hybrid Neural Network-SEIR model for forecasting intensive care occupancy in Switzerland during COVID-19 epidemics
Source: PLoS One. 2022 Mar 3;17(3):e0263789. doi: 10.1371/journal.pone.0263789 (PMC8893679; doi:10.1371/journal.pone.0263789)

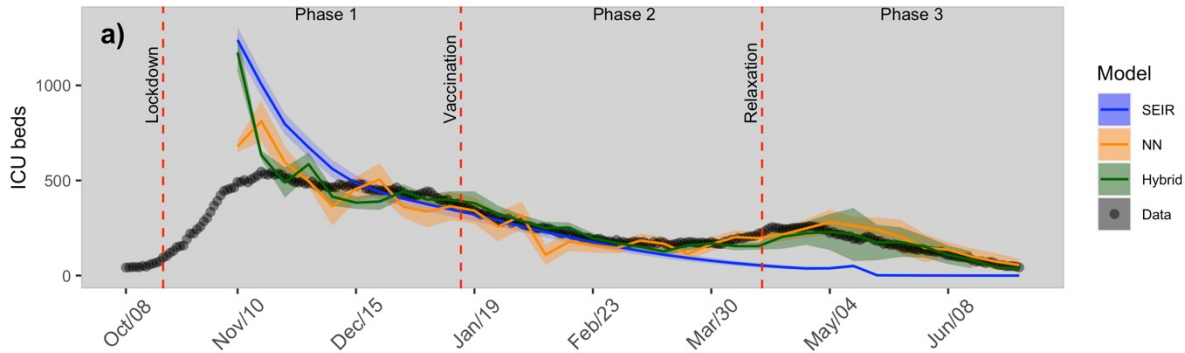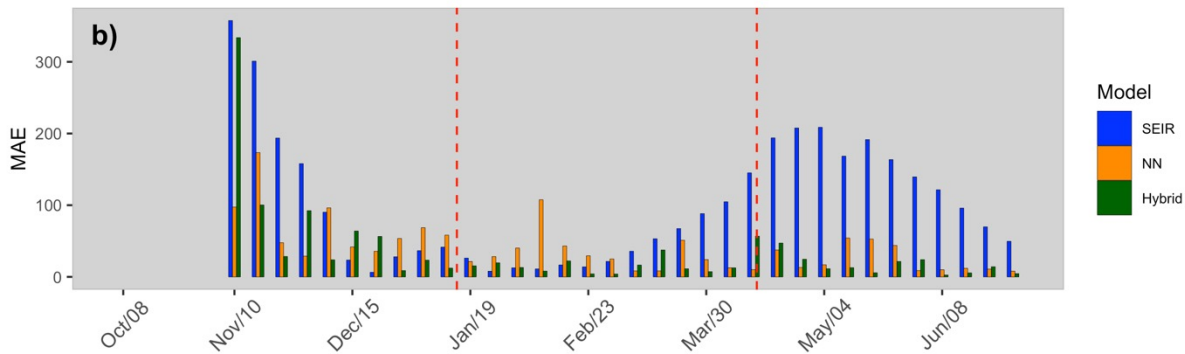

Supplement: S1 Fig — a) Predictions 7-days ahead of intensive occupancy at the national-level for the three models (shaded areas represent 95% confident intervals); b) corresponding Mean Absolute Error (MAE) calculated on test data. (PDF) [file pone.0263789.s001.pdf]

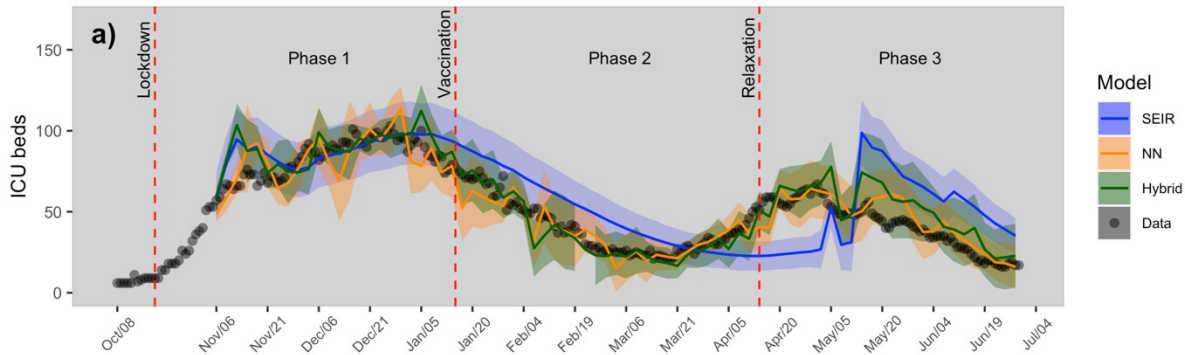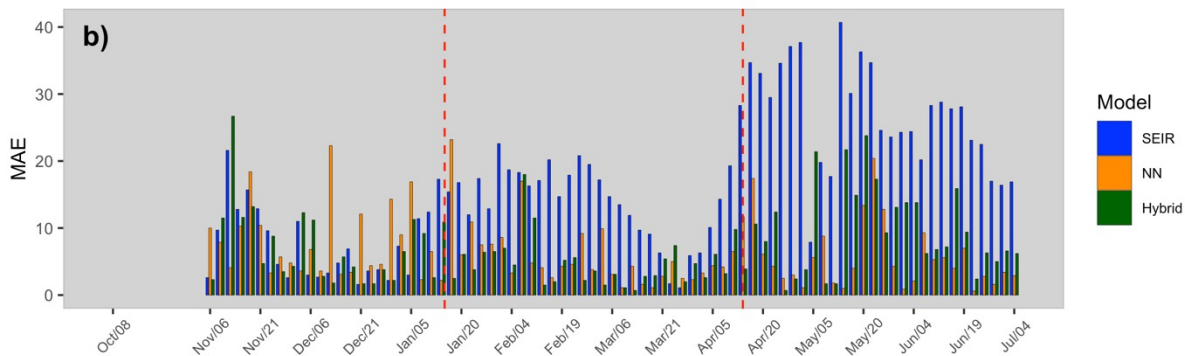

Supplement: S2 Fig — a) Predictions 3-days ahead of intensive occupancy at cantonal level (canton of Zurich) for the three models (shaded areas represent 95% confident intervals); b) corresponding Mean Absolute Error (MAE) calculated on test data. (PDF) [file pone.0263789.s002.pdf]
